# Predictors of Economic Outcomes among Romanian Youth: The Influence of Education—An Empirical Approach Based on Elastic Net Regression

**DOI:** 10.3390/ijerph19159394

**Published:** 2022-07-31

**Authors:** Ana-Maria Zamfir, Adriana AnaMaria Davidescu, Cristina Mocanu

**Affiliations:** 1 Department of Education, Training and Labour Market, National Scientific Research Institute for Labour and Social Protection, 010643 Bucharest, Romania; adriana.alexandru@csie.ase.ro (A.A.D.); mocanu@incsmps.ro (C.M.); 2 Department of Statistics and Econometrics, Bucharest University of Economic Studies, 010552 Bucharest, Romania

**Keywords:** human capital theory, expansion of education, youth, earnings

## Abstract

Young people have to be provided with opportunities to access prosperous, resilient and fulfilling lives. Investing in education and skills is considered one of the most important ways to support young people’s well-being and to enable them to enjoy good career prospects. Using the framework of human capital theory, we explored the role of education among the factors explaining wage variation among Romanian youth. We built our analysis on micro-data for Romania from the EU Statistics on Income and Living Conditions 2020. In order to identify the most important factors influencing the wage distribution, we employed the elastic net regression approach. Moreover, we considered the phenomenon of expansion of education and ran the analysis by alternately using a traditional measure for education and a relative measure reflecting the theory of education as positional good. We ran the analysis for different cohorts of the population, focusing the discussion on the results for young people. Our findings confirm the importance of education for wage distribution together with other factors of influence, such as gender, degree of urbanization, region, sector of employment and working experience. Our conclusions are relevant for designing more effective educational and social policies to deal with various disadvantages faced by youth in Romania.

## 1. Introduction

Successful participation of young people in society and the economy is crucial both for their well-being and to foster economic growth and social inclusion. Well-being incorporates multiple interlinked dimensions, such as health, standard of living, education, political representation and environmental aspects. Recent evidence suggests that well-being dimensions have to be seen more as complements rather than substitutes and that the policies developed have to support a balanced development [1]. While other dimensions of well-being display positive trends in Europe, material well-being remains unchanged [1]. From this point of view, achieving better employment outcomes for young people remains a key objective for public policies and programs in many countries across the world. Moreover, young people are among the groups most vulnerable to economic crises and downturns, experiencing losses in terms of employment [2] and earnings, especially those with low social origins [3] and low education [4]. Difficulties experienced in early careers are linked to negative effects for health and mortality in middle age, including fertility and risky alcohol consumption [5].

Education is beneficial at both the personal and societal levels. Today, schooling is an important agent of socialization, playing a key role in supporting economic, social and personal well-being. Social outcomes of learning include improved physical and mental health and increased social and civic engagement. Existing evidence indicates a statistically significant relation between education and health, and part of this relation is due to the genuine causal role played by higher levels of knowledge and skills [6]. Additionally, education influences the civic engagement and social capital of individuals. Generally, participation in learning is considered a key element in cycles of development and progression [7].

According to human capital theory, individuals decide to acquire more education on the basis of a cost–benefit analysis comparing future returns from additional education with direct and indirect associated costs [8]. This theoretical perspective assumes that the further participation of individuals in education develops their skills, leading to improved work productivity and, subsequently, higher wages [9]. In short, higher expected economic outcomes motivate individuals to pursue additional education [10], and investments in learning are very important paths for human capital development [8]. This theory has been validated by numerous studies showing that higher educational attainment among individuals is associated with better economic outcomes [10,11], including in developing countries [12,13].

Another relevant theoretical perspective is screening theory, which states that education functions as a screening mechanism allowing employers to identify employees with higher abilities who will be given higher wages [9]. Thus, education is one of the most important criteria used by employers for setting wages [14]. Signaling theory provides a complementary perspective, according to which the educational credentials of job seekers signal their abilities [15] and grant them access to specific jobs and wage levels [16]. On the basis of the signaling function of education, better-educated individuals receive higher payments due to the credentials provided by educational attainment [17].

As education grows in importance, many countries are experiencing the phenomenon of expansion of education. According to human capital theory, education can be considered a form of investment in human capital that brings economic returns, both for individuals and societies [18]. On the other hand, in economic theory, some goods have a positional role, meaning that their value is given by their limited supply and due to the fact that they confer a higher position in society to their holders. In the case of positional goods, their distribution is highly relevant for their value. Previously, a number of scholars have questioned the positional character of education and its influence on labour market outcomes [19] and life satisfaction [20]. The positional model assumes that one’s individual position in education distribution is increasingly important in contexts of educational expansion [21]. Moreover, the propensity of education to function as a positional good varies across countries in relation to the institutional context [22].

Studies on the implications of educational expansion for economic returns on education have produced mixed results. Some studies confirmed the positional theory, finding that increasing access and participation to education leads to reduced wage premiums being obtained from education [23]. Other studies concluded that a relative premium remains present in expansion contexts, as poorly educated individuals are those who are penalized on the labour market [24,25]. Moreover, education increases subjective well-being independently of its effect on income, and this effect is stronger when fewer people attain a given level of education, confirming the positional theory [26].

Romania is one of the countries experiencing an ongoing process of educational expansion. Official statistics provided by Eurostat show that the population aged 25–64 years old with tertiary education increased from 14.6% in 2011 to 18.7% in 2020. Participation in higher education increased with greater acceleration among females than males. Furthermore, higher shares of tertiary education have been registered among younger cohorts (in 2020, 25–34 years old: 24.9%, 35–44 years old: 24.6%, 45–54 years old: 15%, 55–64 years old: 9.7%).

**Figure 1:**
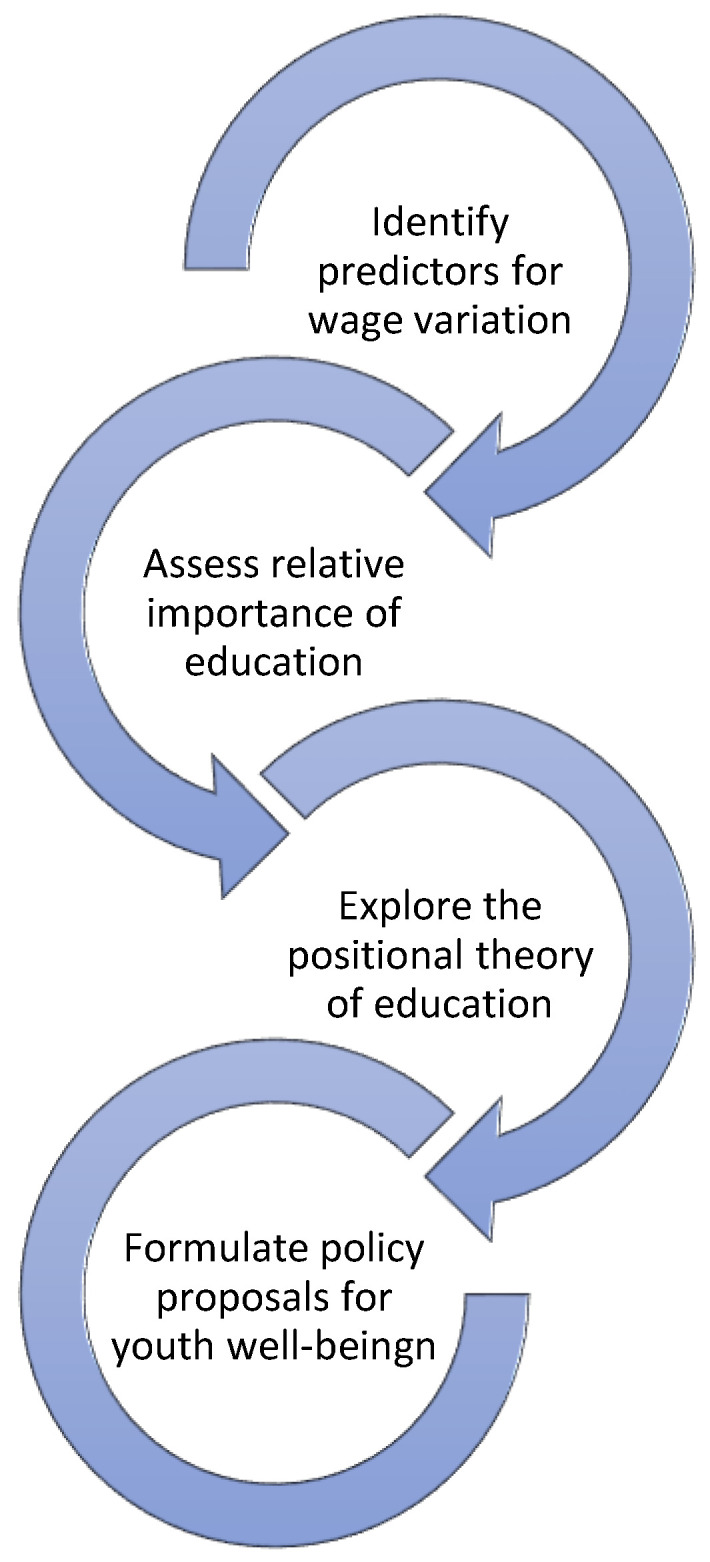
Key objectives of the study.

Using the above theoretical frameworks, this study explored the role of education among the factors explaining wage variation among Romanian youth. We were interested to identify the relative importance of various factors predicting earnings and employed an empirical approach based on elastic net regression. Moreover, we considered the phenomenon of expansion of education and ran the analysis using alternately a traditional measure for education and a relative measure reflecting the theory of education as a positional good. We ran the analysis for different cohorts of the population [27]. We based our analysis on the Mincerian model explaining wage variation as mainly based on the education and working experience of individuals [28]. We explored the importance of the two measures of education for wage distribution, while taking into account other control factors, such as gender, degree of urbanization, region and sector of employment. Our findings are relevant for designing more effective educational and social policies to deal with various disadvantages faced by youth in Romania. The main objectives of the paper are presented in Figure 1.

## 2. Materials and Methods

### 2.1. Study Participants

We built our analysis on micro-data for Romania from Eurostat; namely, the EU Statistics on Income and Living Conditions (EU-SILC) 2020, which includes data on income and living conditions and is based on a standardized methodology applied at European level. This statical survey is one of the main sources for official statistics on income distribution and social exclusion. In addition, this survey collects other data of interest for the present study, such as education, employment and various types of socio-demographic information. We focused our analysis on the dataset for Romania. As the study aimed to explore the influence of education and other predictors for monetary outcomes, we restricted our sample and included working-age full-time employees only (*n* = 5571). 

### 2.2. Variables

The dependent variable measuring the monetary outcomes was the net employee cash income (in local currency). Independent variables included in the study were as follows: education, gender (male, female), degree of urbanization (three categories: densely populated area, intermediate area, thinly populated area), region (NUTS level 1: four macro-regions), working experience and sector of employment. As previous studies found cohort effects in relation to returns on schooling [27], we applied our analysis at the level of age groups: 24 years and younger, 25–34 years, 35–44 years, 45–54 years and 55–64 years old.

In order to explore the theory of education as a positional good, we included in our analysis two alternative measures for education:

1. An absolute measure representing the stock of education: number of years spent in education;
2. A relative measure: educational position of individuals in the general educational distribution (measured as the percentage of individuals with similar or lower educational attainments).

The first variable represents a measure widely used in studies investigating the relation between education and various socio-economic constructs. The second measure reflects the positional theory of education, aiming to explore whether education’s relative importance is conferred by its limited supply.

### 2.3. Statistical Analysis

First, we identified the most important predictors of wage variations. For this purpose, we employed elastic net regression (Enet). This method allows the selection of the most relevant variables for the analyzed outcome [29]. Elastic net regression is a combination of two widely used, regularized linear regression methods: ridge and lasso. Similar to lasso, Enet achieves automatic variable selection and continuous reduction while carrying out the selection of groups of associated variables [30,31]. Enet reduces the regression coefficients:

(1) $${\hat{\beta }}_{enet}=\left(1+\frac{\lambda_{2}}{n}\right)\left\{\arg\underset{\beta }{\min}∥y-\sum_{j=1}^{p}x_{j}\beta_{j}{∥}^{2}+\lambda_{1}{∥\beta ∥}_{1}+\lambda_{2}{∥\beta ∥}_{2}^{2}\right\}$$

In this way, we created the conditions for an improved earnings model to be applied in the second step by including only control variables identified as relevant through the Enet method. Thus, we further applied a Mincer model, which is an earnings function widely used to estimate the returns on education [32]. The Mincer equation used was as follows:

(2) $$W_{i}=\beta_{0}+\beta_{1}S_{i}+\beta_{2}X_{i}+\beta_{3}X_{i}^{2}+control\;variables+\varepsilon$$

where $W_{i}\;$ is the wage of a person *i*, $S_{i}$ is the education variable, $X_{i}\;$ is the number of years spent on the labour market and the control variables are region, sector, degree of urbanization and gender.

As well as education, the Mincer equation includes work experience as an important explanatory variable representing the accumulation of human capital post-education [28]. This is an important aspect, as previous studies have shown that, by not including work experience in the model, the effects of schooling on earnings can be underestimated [33].

## 3. Results

**Figure 2:**
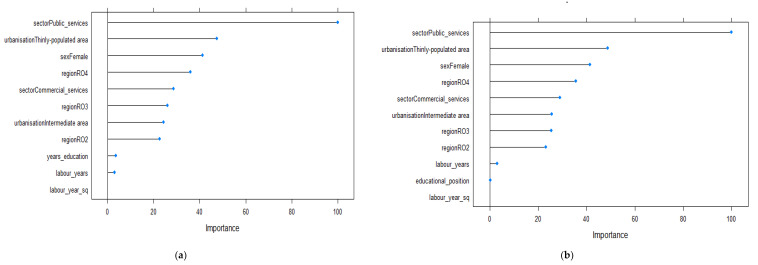
Relevant variables in explaining the variations in wages based on the Enet method for the 24 years old and younger age group (*n* = 155): (**a**) model 1 using the absolute measure of education; (**b**) model 2 using the relative measure of education.

Figure 2 presents the Enet results showing the relative importance of various predictors for explaining the cash income variation in the 24 years old and younger age group. The results were similar for both models: model 1 using the absolute measure of education (Figure 2a) and model 2 using the relative measure of education (Figure 2b). For this age group, sector of employment, degree of urbanization and gender were the most important factors influencing monetary outcomes. The wage model estimations (Table A1) indicated that working in public services is positively associated with higher wages. The second important predictor was the gender of young people. Our results showed that being female decreases the wage level. In addition, living in thinly populated areas is negatively associated with increased monetary outcomes. Furthermore, for youth under 24 years old, the region in which they live influenced their wage level in a significant manner. Living in RO4 and RO2 regions was associated with higher wages, while living in an RO3 region was associated with lower monetary outcomes. As expected for this age category, education, both in absolute and relative terms, as well as working experience had low influences on wage variation.

**Figure 3:**
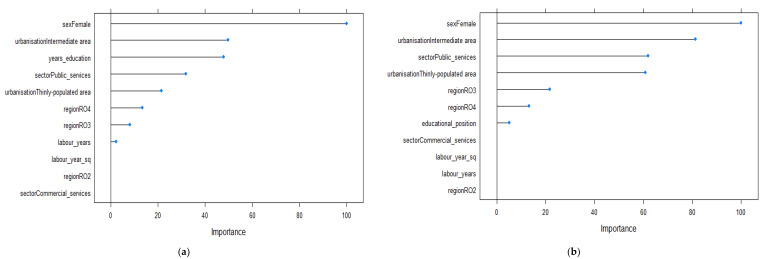
Relevant variables in explaining the variations in wages based on the Enet method for the 25–34 year old age group (*n* = 947): (**a**) model 1 using the absolute measure of education; (**b**) model 2 using the relative measure of education.

Figure 3 presents the relative importance of the factors predicting wage level for the 25–34 year old age group. When using the absolute measure of education, gender, degree of urbanization and number of years spent in education were the most important factors shaping the economic outcomes of individuals (Figure 3a). Thus, being a female and living in an intermediately populated area were associated with lower wages. On the other hand, the higher the number of years spent in education, the higher the economic outcomes were found to be. When using educational position as a predictor, the relative importance of education decreased and working in public services and the region of residence became more influential (Figure 3b). Other factors that positively influenced the wage level were working in public services and living in an RO3 region. In contrast, residing in a thinly populated area or in an RO4 region decreased economic outcomes among the 25–34 year old age group.

**Figure 4:**
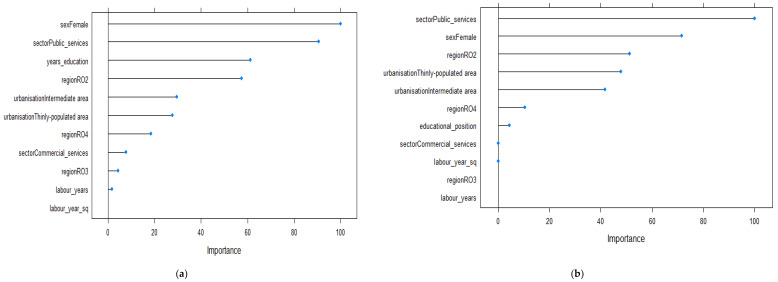
Relevant variables in explaining the variations in wages based on the Enet method for the 35–44 year old age group (*n* = 1568): (**a**) model 1 using the absolute measure of education; (**b**) model 2 using the relative measure of education.

The relative importance of factors influencing wages for the 35–44 year old age group is indicated in Figure 4. When using the number of years in schooling, education was found to be the third most important predictor for wage variation, after gender and working in public services (Figure 4a). Thus, being female decreased the wage level, while working in public services had a positive influence on monetary outcomes. On the other hand, position held in the education distribution was not as important as the years of education, and region and degree of urbanization have stronger influences on economic returns (Figure 4b). Estimations showed that living in thinly or intermediately populated areas, as well as in RO2 or RO4 regions, had negative effects on wages (Table A2).

**Figure 5:**
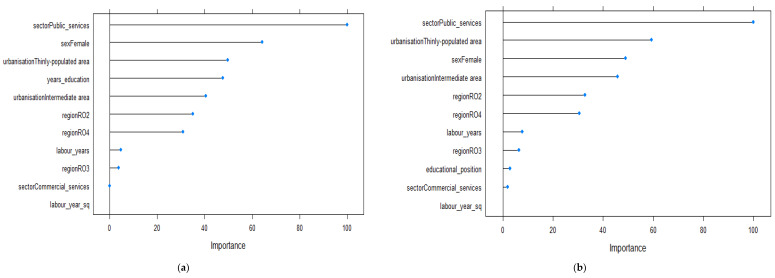
Relevant variables in explaining the variations in wages based on the Enet method for the 45–54 year old age group (*n* = 2033): (**a**) model 1 using the absolute measure of education; (**b**) model 2 using the relative measure of education.

Figure 5 displays the relative importance of the factors predicting economic outcomes in the case of the 45–54 year old age group. First, working in public services was the most important factor conducive to higher wages. Less important but of influence were being female and living in an area with low population density (Figure 5a). Both characteristics were negatively associated with increased economic outcomes. In addition, a higher number of years in education increased wage levels. Less influentially, living in an intermediately populated area or in RO2, RO4 or RO3 regions had negative effects on wages. Furthermore, years of working experience had a small but positive influence on wages. On the other hand, the educational position of individuals was less essential for predicting economic outcomes (Figure 5b).

**Figure 6:**
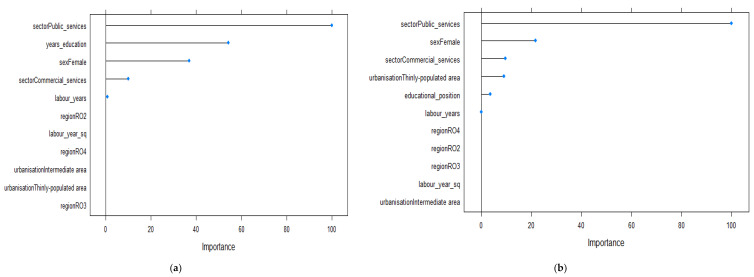
Relevant variables in explaining the variations in wages based on the Enet method for the 55–64 year old age group (*n* = 868): (**a**) model 1 using the absolute measure of education; (**b**) model 2 using the relative measure of education.

Figure 6 presents the relative importance of predictors for the 55–64 year old age group. In this case, the number of years of education was the second most important predictor of wage level, after being employed in the public sector. Being female and working in commercial services had negative but more reduced influences on wages (Figure 6a). This was the only age group for which the influence of the absolute measure of education was more important than gender for predicting the wage level. Further, educational position was less important for predicting levels of economic outcomes. When taking into consideration the positional character of education, living in thinly populated areas or working in commercial services became more important and displayed a negative effect on monetary outcomes (Figure 6b).

## 4. Discussion

For all age groups, education was positively associated with higher wages. Both the absolute level of education and its level relative to others had positive and significant relations with the wage level. Therefore, investing in education is one of the most important ways to obtain higher economic outcomes, including for young people. Education ad a higher influence on wage level after the age of 25 years old, confirming the human capital theory according to which individuals decide to invest in additional education by postponing present benefits in favor of future higher earnings [8,18].

Our results showing the positive influence of education on earnings are consistent with findings from other studies indicating positive returns on schooling in both developed and developing countries [29]. Romania matches the profile of upper-middle- and high-income economies in which private returns are high, especially for tertiary education [34]. According to previous comparable estimates, the returns on one additional year of education peak in Sub-Saharan Africa and register the lowest levels in the Middle East/North Africa region [35]. Eastern European countries, including Romania, display below-average returns [35].

Comparing the results obtained by using the two alternative measures of education, we found that the theory of the positional character of education was not very representative for the Romanian context, including for the younger population. The absolute stock of education remained much more important in predicting the wage level, while the level of education relative to others was less important in this respect. Our results showed that, in the Romanian case, educational expansion is highly beneficial for young people as it enhances their access to good career prospects and higher wage levels. Thus, this study complements the existing literature, which has provided evidence on the question of whether educational expansion impacts returns on schooling. In this respect, our analysis found no clear evidence that the expansion of education influences economic returns, confirming the conclusions of previous analysis on developing countries from Africa, Latin America, Asia and Eastern Europe [12]. While studies providing comparable cross-country estimates found that there is a general, modestly declining pattern in returns on education as the availability of schooling increases [35], others showed that Eastern Europe is the only region displaying a different trend in recent decades [12]. Such an evolution is explained by the low rates of returns in the years of the post-communist transition, followed by increasing returns fueled by the rising demand for human capital due to accelerated economic growth after the year 2000 [12].

On the other hand, the reduced level-of-experience wage premium for accumulated labour market experience shows that Romania is more similar to countries where job tenure and firm-specific skills are better rewarded than general labour market skills.

Although regional wage variation is significant in Romania, the number of years spent in education by young people is more influential for their monetary outcomes than their region of residence. However, other factors, such as gender and degree of urbanization, have higher influences on young people’ wages than education. Our results are consistent with official statistics that indicate a persistent gender pay gap in Europe, including in Romania. Similarly to other European countries, the gender pay gap in Romania is lower among new entrants to the labor market and tends to widen with age. Career interruptions experienced by women due to family reasons are one of the causes explaining this age-related trend. Moreover, together with Poland, Romania displays a specific pattern whereby gender becomes less influential for earnings after the age of 55. In addition, our results showed that education only has more influence than being female after the age of 55, when family responsibilities dwindle.

Furthermore, thinly and intermediately populated areas, covering rural and small urban localities with poor economic development, offer limited well-paid employment opportunities for young people. Romania is one of the European countries with the highest share of rural population at risk of poverty and social exclusion. While the demand for human capital is low in such areas, the return on education remains less important. High urban–rural disparities in earnings in European countries, including Romania, can be explained by the low density of the rural population, the high proportion of farmers, the post-socialist transition and the generally lower economic development [36].

These results reveal important vulnerabilities and call for the construction of integrated youth policies around measures supporting education expansion while simultaneously tackling such structural disadvantages affecting employment outcomes.

## 5. Policy Implications

Our results stress the importance of implementing more effective and inclusive educational policies to improve the chances of all young people to lead prosperous, resilient and fulfilling lives. On the one hand, policy interventions should address the various obstacles preventing individuals from further investing in education in order to alleviate existing constrains. First, programs aiming to reduce the number of students leaving education early need to improve their impact, especially by preventing school dropouts in thinly populated areas. In this regard, financial support for disadvantaged students, together with programs providing school lunches and after-school activities, would help reduce absenteeism and dropout rates in primary and secondary education. Moreover, policies have to provide better incentives and support to expand participation in higher education, including among under-represented groups, such as the rural population. The agenda for widening participation in education has to be promoted by enhancing the support measures granted for under-represented students, mainly by providing financial support for transport, housing and educational materials, as well as funding mechanisms based on granting individual vouchers for education or scholarships to those with low incomes. An expansion of cost-sharing and financing mechanisms, such as flexible student loans, could respond to the increasing demand for higher education in the context of high returns on university degrees. Furthermore, implementing effective early-warning systems for students at risk of dropping out would be highly beneficial at all educational levels. Such policy interventions would alleviate relevant constraints and contribute to risk adjustment for those investing in education. In this way, education would become a more attractive investment, allowing more people to collect positive returns from education. On the other hand, an education system that is more effective in improving cognitive and non-cognitive skills for all young people would contribute to equity and efficiency in the long term. Thus, expansion of tertiary education should not be promoted by policy makers to the detriment of basic education. Furthermore, the access to and quality of secondary education for students from isolated and rural areas could be improved through tailor-made delivery models, vouchers and scholarships. The extension of the current programs of dual education, together with support measures for students who commute from rural and remote areas, could increase participation in upper secondary education among disadvantaged students. In addition, policy interventions supporting school-to-work transitions, such as work-based learning arrangements, could facilitate the smoother and faster transition of graduates to stable employment. Educational programs that allow students to work part-time would reduce the opportunity costs of higher education, improving practical skill development and economic outcomes among young people.

Furthermore, encouraging the participation of girls and women in all educational fields would contribute, in the long term, to a reduction in the gender pay gap. Maintaining efforts to invest in girls’ educations at all levels would produce overall efficiency gains and reduce the gender gap. In addition, improved counselling and guidance services for students and school leavers would help support them in making better educational and career decisions that maximize their potential and produce long-term effects on their well-being. From a macro perspective, an improved system providing information on the returns from various educational tracks would accelerate the process through which educational supply responds to the labor market demand for educated workers.

On the other hand, economic policies supporting a more balanced territorial development would improve employment opportunities, supporting poverty reduction and social inclusion in under-developed areas with long-term benefits for individuals and communities. In addition, employment policies have to support better allocation of job seekers on the labor market, especially for women and people from rural areas or under-developed regions. Active labor market policies have to improve access among job seekers to quality employment, including by commuting or through geographical mobility, as well as training and retraining programs for qualifications in demand.

As the economic outcomes of young people are affected by various structural vulnerabilities, a dedicated tax deduction could be an effective way to increase earnings among youth. While taxation progressivity has been found to be rather detrimental for the incentive to study [37], tax reductions for young people would increase monetary outcomes for them while keeping education attractive.

In addition, wider programs to help in early childhood and child care-services and life–work balance would improve the participation of women in the labor market, including in well-paid jobs and top positions. Increasing accessibility through flexible work arrangements would support young women’s participation in the labor market. Moreover, enhancing systems of collective regulation would improve gender equality in terms of wages.

Our results confirm that education is a key factor in cycles of development and progression, bringing long-term benefits for individuals and society. Continuing the efforts of the Romanian government to further increase educational attainment would be highly beneficial for all the interlinked dimensions of well-being, such as standard of living, health, political representation and environmental protection.

## 6. Conclusions

This study addressed a research gap concerning the insufficient consideration of education as a positional good in the literature so far [38]. We found no clear evidence supporting the positional model of education and conclude that education expansion remains one of the most important ways of improving economic outcomes for Romanian youth. Our results have implications that should encourage policymakers to expand education even more in the future in order to increase educational attainment among young cohorts. In addition to educational expansion, measures for reducing the gender pay gap and rural–urban disparities should be included in youth agendas in the years to come.

## Data Availability

Data are available from Eurostat according to the Commission Regulation (EU) No 557/2013 of 17 June 2013 implementing Regulation (EC) No 223/2009 of the European Parliament and of the Council on European Statistics as regards access to confidential data for scientific purposes and repealing Commission Regulation (EC) No 831/2002.

## Appendix A

**Table A1:** Coefficients for model 1 using the absolute measure of education.

	24 Years and Younger	25–34	35–44	45–54	55–64
**Intercept**	4202.779	1295.033	−174.576	−611.590	−2503.705
**Sex (female)**	−517.633	−741.809	−827.339	−791.740	−431.974
**Years in education**	47.769	355.357	506.340	586.833	635.458
**Urbanization (intermediate area)**	−306.264	−369.423	−244.567	−498.086	
**Urbanization (thinly populated area)**	−594.827	−159.444	−228.995	−612.424	
**Region RO2**	283.785		−475.758	−432.339	
**Region RO3**	−327.481	60.604	−36.683	−47.895	
**Region RO4**	452.061	−99.022	−152.522	−382.060	
**Sector (commercial services)**	−358.932		−64.799	−1.918	−117.399
**Sector (public services)**	1249.145	236.477	749.157	1230.946	1171.564
**Labor years**	41.611	16.622	13.973	58.411	9.849
**Labor years squared**	−2.632			−1.094	

**Table A2:** Coefficients for model 2 using the relative measure of education.

	24 Years and Younger	25–34	35–44	45–54	55–64
**Intercept**	4426.443	3222.834	2735.201	1135.956	818.908
**Sex (female)**	−519.707	−684.348	−776.176	−813.637	−352.952
**Educational position**	4.632	34.440	46.885	51.671	59.109
**Urbanization (intermediate area)**	−323.657	−556.482	−451.883	−760.666	
**Urbanization (thinly populated area)**	−611.733	−415.969	−520.057	−985.146	−147.383
**Region RO2**	292.311		−555.267	−545.786	
**Region RO3**	−320.519	148.647		−10.512	
**Region RO4**	446.714	−90.945	−113.098	−507.047	
**Sector (commercial services)**	−365.904		−0.583	−33.800	−157.909
**Sector (public services)**	1251.546	424.535	1085.787	1659.223	1635.185
**Labor years**	40.562			131.617	0.345
**Labor years squared**	−2.544		−0.161	−3.010	
